# Binding, Transcytosis and Biodistribution of Anti-PECAM-1 Iron Oxide Nanoparticles for Brain-Targeted Delivery

**DOI:** 10.1371/journal.pone.0081051

**Published:** 2013-11-20

**Authors:** Mo Dan, David B. Cochran, Robert A. Yokel, Thomas D. Dziubla

**Affiliations:** 1 Department of Pharmaceutical Sciences, College of Pharmacy, University of Kentucky, Lexington, Kentucky, United States of America; 2 Graduate Center for Toxicology, University of Kentucky, Lexington, Kentucky, United States of America; 3 Chemical and Materials Engineering, University of Kentucky, Lexington, Kentucky, United States of America; Case Western Reserve University, United States of America

## Abstract

**Objective:**

Characterize the flux of platelet-endothelial cell adhesion molecule (PECAM-1) antibody-coated superparamagnetic iron oxide nanoparticles (IONPs) across the blood-brain barrier (BBB) and its biodistribution *in vitro* and *in vivo*.

**Methods:**

Anti-PECAM-1 IONPs and IgG IONPs were prepared and characterized in house. The binding affinity of these nanoparticles was investigated using human cortical microvascular endothelial cells (hCMEC/D3). Flux assays were performed using a hCMEC/D3 BBB model. To test their immunospecificity index and biodistribution, nanoparticles were given to Sprague Dawley rats by intra-carotid infusion. The capillary depletion method was used to elucidate their distribution between the BBB and brain parenchyma.

**Results:**

Anti-PECAM-1 IONPs were ∼130 nm. The extent of nanoparticle antibody surface coverage was 63.6±8.4%. Only 6.39±1.22% of labeled antibody dissociated from IONPs in heparin-treated whole blood over 4 h. The binding affinity of PECAM-1 antibody (K_D_) was 32 nM with a maximal binding (B_max_) of 17×10^5^ antibody molecules/cell. Anti-PECAM-1 IONP flux across a hCMEC/D3 monolayer was significantly higher than IgG IONP's with 31% of anti-PECAM-1 IONPs in the receiving chamber after 6 h. Anti-PECAM-1 IONPs showed higher concentrations in lung and brain, but not liver or spleen, than IgG IONPs after infusion. The capillary depletion method showed that 17±12% of the anti-PECAM-1 IONPs crossed the BBB into the brain ten minutes after infusion.

**Conclusions:**

PECAM-1 antibody coating significantly increased IONP flux across the hCMEC/D3 monolayer. *In vivo* results showed that the PECAM-1 antibody enhanced BBB association and brain parenchymal accumulation of IONPs compared to IgG. This research demonstrates the benefit of anti-PECAM-1 IONPs for association and flux across the BBB into the brain in relation to its biodistribution in peripheral organs. The results provide insight into potential application and toxicity concerns of anti-PECAM-1 IONPs in the central nervous system.

## Introduction

Multifunctional superparamagnetic iron oxide nanoparticles (IONPs) have various applications, such as diagnosis and therapy of the central nervous system (CNS) [1], [2]. For example, IONPs have drawn increasing attention as T2 magnetic resonance imaging (MRI) contrast agents in preclinical and clinical studies to evaluate blood-brain barrier (BBB) dysfunction related to tumors and other pathologies such as stroke and carotid atherosclerosis [3], [4]. Multifunctional IONPs also provide the possibility to deliver therapeutic agents to the brain and concurrently monitor their tissue distribution using MRI [5], [6]. One of the challenges for CNS applications of IONPs is the ability to cross the highly restricted BBB. Previous research has suggested evidence of IONP flux across the BBB by analyzing the whole brain concentration. However, there have been no reports distinguishing between IONPs in the brain vessels and BBB cells [7]; or they only showed the therapeutic efficacy of co-delivered compounds in animal models of brain tumors [8]. To advance the potential applications of multifunctional IONPs in the CNS, there is an urgent need to understand how they associate with, and transcytose across, the BBB *in vitro* and *in vivo*.

Brain capillary endothelial cells cooperate with pericytes, astrocytes, and neurons to generate and maintain the unique barrier properties of the BBB. The BBB plays a crucial role in safeguarding the brain from endogenous and exogenous compounds, which includes most therapeutics [9]. A recent study evaluated the uptake and flux of IONPs using human brain-derived endothelial cells. Flux of IONPs that lacked targeting moieties on the surface was very limited under normal conditions [10]. A promising strategy to enhance IONP flux across the BBB is to use a BBB targeting moiety. Platelet-endothelial cell adhesion molecule (PECAM-1) (CD31) is a member of the immunoglobulin superfamily that is constitutively expressed on endothelial cell membranes and is involved in transcytosis of activated leukocytes across the BBB in neuroinflammation [11], [12], [13]. Furthermore, significant upregulation of PECAM-1 in neuroinflammation provides a potential to target the CNS for the treatment of neurological conditions such as stroke and brain tumor [13]. Pure PECAM-1 antibody has been shown to target the endothelial lumen, but does not internalize into endothelial cells [14]. However, anti-PECAM-1 coated nanocarriers can enter the endothelial cells through a unique vesicular internalization pathway, cell adhesion molecule (CAM)-mediated endocytosis [15]. Anti-PECAM-1 antibodies conjugated to diverse therapeutic cargoes and nanocarriers provided robust intracellular drug delivery into endothelial cells [16]. However, how anti-PECAM-1 nanocarriers associate with and traffic across the BBB, one of the most important endothelial cell barriers, still needs to be defined.

We hypothesized that PECAM-1 antibody will increase IONP BBB association, trafficking across the BBB, and change its distribution profile *in vitro* and *in vivo*. In this study, we characterized anti-PECAM-1 IONPs for size, stability in blood, and association to immortalized human cortical microvascular endothelial (hCMEC/D3) cells. We investigated the association, and flux across the BBB over 6 h, of anti-PECAM-1 IONPs using hCMEC/D3 cells. Furthermore, anti-PECAM-1 IONP brain accumulation and biodistribution in peripheral organs were studied in Sprague Dawley rats. The capillary depletion method was used to test anti-PECAM-1 IONP *in vivo* distribution between the BBB endothelial cells and brain parenchyma. The results of this study demonstrate the potential of anti-PECAM-1 IONPs to target and transcytose across the BBB and enter into the brain parenchyma, providing valuable insight into the feasibility of anti-PECAM-1 IONP as a brain targeting MRI contrast agent and/or drug delivery system for the CNS.

## Materials and Methods

### Ethics Statement

This study used 21 male Sprague-Dawley rats, weighing 300±25 g (mean ± SD), that were housed individually prior to study in the University of Kentucky Division of Laboratory Animal Resources Facility under a 12∶12 h light∶dark cycle at 70±8°F and 30 to 70% humidity. The rats had free access to 2018 Harlan diet and reverse osmosis water. Animal work was approved by the University of Kentucky Institutional Animal Care and Use Committee (Protocol 2008-0272). The research was conducted in accordance with the Guiding Principles in the Use of Animals in Toxicology.

### Reagents

Mouse anti-human anti-PECAM-1 was created and purified in house through the use of a hybridoma cell line (P2B1) purchased from the Developmental Studies Hybridoma Bank (Iowa City, IA). For *in vivo* studies, a mouse anti-rat anti-PECAM-1 (Clone TLD-3A12) was purchased from Millipore (Billerica, MA). Nonspecific mouse IgG was from Jackson Immuno (West Grove, PA). Na^125^I was purchased from Perkin Elmer (Boston, MA). All other reagents were from Sigma-Aldrich (St. Louis, MO).

### Iron oxide nanoparticle synthesis

IONPs were synthesized using a previously reported method [17]. Briefly, ferric chloride hexahydrate (Fe^3+^) and ferrous chloride tetrahydrate (Fe^2+^) were dissolved in deionized water (Fe^3+^∶Fe^2+^ = 2∶1), followed by adding ammonium hydroxide dropwise under an N_2_ atmosphere at 85°C. After 1 h, the solution was placed on a magnet to collect black brown particles, which were washed repeatedly using pure ethanol [18], [19]. IONPs were dried overnight in a vacuum drying oven. Properties, such as size and zeta potential of the IONPs, were determined in our laboratories. All of the methods have been previously reported [20].

### Protein iodination for antibody tracing

IgG and the anti-PECAM-1 antibody were labeled with Na^125^I using the Iodogen method. In brief, 100 µg of antibody was mixed with 15 µCi of Na^125^I for 5 minutes in glass tubes coated with Iodogen reagent. Following the reaction, the now-labeled protein was purified using Bio-Rad Labs packed spin columns (Hercules, CA). The extent of iodination was determined by protein precipitation followed by analysis of radioactivity in the pellet and supernatant [21].

### Preparation and characterization of antibody-modified iron oxide surfaces

Iron oxide nanoparticles were suspended in PBS and sonicated with a probe sonicator at a power output of 10 W for 1 minute, then transferred to a sonication bath for 30 minutes prior to surface coating. To couple anti-PECAM-1 or IgG antibody to iron oxide nanoparticles, a physioabsorption technique was employed. Either anti-PECAM-1 or IgG antibodies were incubated with the nanoparticles at a solution concentration equivalent to 10,000 antibodies/µm^2^ particle surface area, 1.2 times the theoretical monolayer coating based on antibody size and particle surface area. Radiolabeled anti-PECAM-1 or IgG in PBS was added to the suspended nanoparticles and incubated for 1 h at 25°C. Particles were washed with PBS 3 times and centrifuged for 30 minutes at 22,000 *g* and then suspended in 1% BSA-PBS. The antibody was traced in both supernatant and pellets using a PerkinElmer 2470 Automatic Gamma Counter to determine the extent of surface coverage.

### Antibody-modified iron oxide nanoparticle stability in whole blood

To determine coating stability *in vivo*, particles were incubated in heparin-treated whole rat blood at 37°C for 24 h at equivalent concentrations utilized *in vivo* (0.015 mg nanoparticles/ml whole blood). At pre-determined time points, aliquots of whole blood were centrifuged for 30 minutes at 22,000 *g* and separated from the nanoparticles and analyzed on the gamma counter.

### Cell lines and culture conditions

hCMEC/D3 cells were obtained under license from INSERM, France. The cells were maintained in endothelial growth medium-2 supplemented with 2.5% fetal bovine serum, 1% penicillin and streptomycin, 0.1% fibroblast growth factor, 0.01% hydrocortisone and 0.025% vascular endothelial growth factor, insulin-like growth factor and endothelial growth factor, under 37°C and 5% CO_2_. Cells were passaged into collagenated culture flasks every 3–4 days when they reached approximately 85%–95% confluence [22], [23], [24].

### PECAM-1 antibody binding affinity to hCMEC/D3 cells

hCMEC/D3 cells were seeded on 36 well plates at a density of 50,000 cells/cm^2^. They were incubated with serial dilutions from 0.78 to 100 nM of ^125^I anti-PECAM-1 or ^125^I IgG antibodies for 2 h (n = 3). The supernatants in the donor chamber were collected and the cells were washed 3 times with PBS at 4°C. The hCMEC/D3 monolayer was lysed with 1% Triton X-100 in 1.0 N NaOH. The cell lysate and supernatant (including the washing solution) radioactivities were measured using a Wallac 1470 Wizard™ gamma counter. B_max_ and K_D_ were calculated using GraphPad Prism (GraphPad Software, San Diego, CA, USA).

### Anti-PECAM-1 IONP cell association and flux using the human brain endothelial cell line hCMEC/D3 in vitro BBB model

hCMEC/D3 cells were seeded on type I collagen pre-coated 6 well Transwell filters (polycarbonate 12 mm, pore size 3.0 µm) at a density of 50,000 cells/cm^2^. Flux assays were performed 7-10 days after seeding [22], [23]. The tightness of the hCMEC/D3 monolayer was measured as transepithelial electrical resistance (TEER) using a RMA321-Millicell-ERS voltohmmeter (Millipore Corp, Billerica, MA). To monitor flux through the paracellular pathway, lucifer yellow (LY, 100 µM) was added to the medium on the donor side of the cells. Samples of the medium from the donor chamber were collected at time zero and from the receiving chamber hourly for 6 h for LY concentration analysis. LY fluorescence was determined in a SpectraMax M5 Multi-Mode Microplate Reader (Molecular Devices, Sunnyvale, CA) at λ_ex_\λ_em_ = 450/530 nm and compared with a standard of LY in endothelial growth medium-2.

Anti-PECAM-1 and IgG IONPs were introduced into the donor chamber at 0.05 mg/mL, as used in our previous cytotoxicity study on IONPs [20]. Samples (100 µL) were collected from the donor chamber at time 0 and the receiving chamber hourly for 6 h for iron concentration analysis by inductively coupled plasma mass spectrometry (ICP-MS) (Agilent 7500cx, Santa Clara, CA, USA). The donor chamber was removed and the cells washed 3 times using PBS at 4°C. The hCMEC/D3 monolayer was lysed with 1% Triton X-100 in 1.0 N NaOH. Iron concentration in the cell lysate and supernatant (including washing solution) was measured using ICP-MS. Flux rates of LY and nanoparticles were calculated by linear regression for the first 6 h. Two-way ANOVA followed by Bonferroni multiple comparisons was used to test for significant flux differences among the treatment groups and times using GraphPad Prism (GraphPad Software, San Diego, CA). Statistical significance was accepted at p<0.05.

### Brain targeting and biodistribution by anti-PECAM-1 IONPs

Antibody-coated iron oxide nanoparticles were prepared similarly as before with one exception. Both anti-PECAM-1- and IgG-coated particles were incubated with 5% ^125^I labeled IgG at a concentration of 10 mg/ml. This was done to prevent any detached labeled antibody from accumulating in the vasculature, thus providing a false positive for adhesion. Carotid artery injection was employed to delivery 10 mg/kg ^125^I anti-PECAM-1 IONPs (n = 3) and ^125^I IgG IONPs (n = 3). Briefly, the rat was anesthetized under ketamine/xylazine anesthesia (75 and 5 mg/kg), and its left carotid artery exposed. Following ligation of the external carotid, occipital and common carotid arteries, PE60 tubing containing heparin (100 U/ml, in 0.9% NaCl) was inserted into the common carotid. The 10 mg/ml ^125^I anti-PECAM-1 IONPs and ^125^I IgG IONPs were infused in 1 mlover 1 min at a dose of 10 mg/kg. All the rats were sacrificed 10 min after infusion. The brain was harvested and cleaned of meninges and surface vessels. Blood and organs such as the liver, spleen and lung were collected for biodistribution analysis using the gamma counter. The results were compared between ^125^I anti-PECAM-1 and ^125^I IgG IONPs using t-test or one-way ANOVA. The localization ratio (LR) was calculated as the percent of the injected dose per gram of tissue divided by percent of the injected dose per gram of blood. The specificity index was calculated as the LR of the targeted formulation (anti-PECAM-1 IONPs) divided by the non-targeted counterpart (IgG IONPs). The specificity index indicates specific targeting to organs, normalized by organ weights and the fraction contained in blood [25]. One-way ANOVA followed by Tukey's test was used to test for significant differences of IgG IONP and anti-PECAM-1 IONP biodistribution among different organs. All results are reported as mean ± SD. Statistical significance was accepted at p<0.05.

### BBB integrity assessment

Five minutes before termination, the rat was given 6 mg Na fluorescein (334 Da) interartery in 1 ml saline over 40 s as a BBB permeability marker. Postmortem brain cortex was obtained to quantify fluorescein content. Fluorescence was determined in a SpectraMax M5 Multi-Mode Microplate Reader (Molecular Devices, Sunnyvale, CA) at λ_ex_\λ_em_ = 493/514 nm. The results among control, IgG IONP, and anti-PECAM-1 IONP groups were compared using one-way ANOVA (GraphPad Prism).

### Anti-PECAM-1 IONP distribution between the BBB endothelial cells and brain parenchyma using the capillary depletion method

The capillary depletion method was used to separate brain parenchyma from capillary tissue [26], [27]. After a 10 mg/kg ^125^I anti-PECAM-1 IONP or ^125^I IgG IONP injection into the left carotid artery, a 20 s washout was conducted using PBS at a flow rate of 20 ml/min immediately before decapitation [28]. The forebrain from the left hemisphere was isolated from ^125^I anti-PECAM-1 IONP and ^125^I IgG IONP treated rats (n = 3) and the lateral ventricle choroid plexus in the perfused hemisphere removed. The tissue was homogenized in 3.5 ml of buffer containing 141 mM NaCl, 4 mM KCl, 2.8 mM CaCl_2_, 1 mM NaH_2_PO_4_, 1 mM MgSO_4_, 10 mM glucose and 10 mM HEPES at pH 7.4. Dextran (70,000 g/mol) was then added to 18% (w/v) and the sample further briefly homogenized. After centrifugation at 5400× g for 15 min at 4°C, the supernatant (brain rich fraction) and pellet (capillary rich fraction) were carefully separated for measurement of ^125^I by gamma counter. The percentage of the forebrain ^125^I in the capillary rich fraction was calculated as follows: (Mass of ^125^I in capillary rich fraction/Mass of ^125^I in capillary rich fraction and capillary depleted fraction)*100

### The apparent permeability coefficient

The apparent permeability coefficients (P_app_, cm/s) of LY, IgG IONPs and anti-PECAM-1 IONPs were calculated using GraphPad Prism. The first 6 h flux data were used with R^2^ cutoff >0.8. The P_app_ was calculated using the equation: P_app_ = (ΔQ/Δt)/(area*C_D_) [29]. *ΔQ/Δt* is the linear appearance rate obtained from the profile of the transported amount of the substrate against time (mg/s). *C_D_* is the initial donor concentration of LY or nanoparticles (mg/cm^3^). *Area* is the surface area of the cell monolayer (4.67 cm^2^ for a 6-well plate).

## Results

### Anti-PECAM-1/IgG coating efficiency

**Figure 1:**
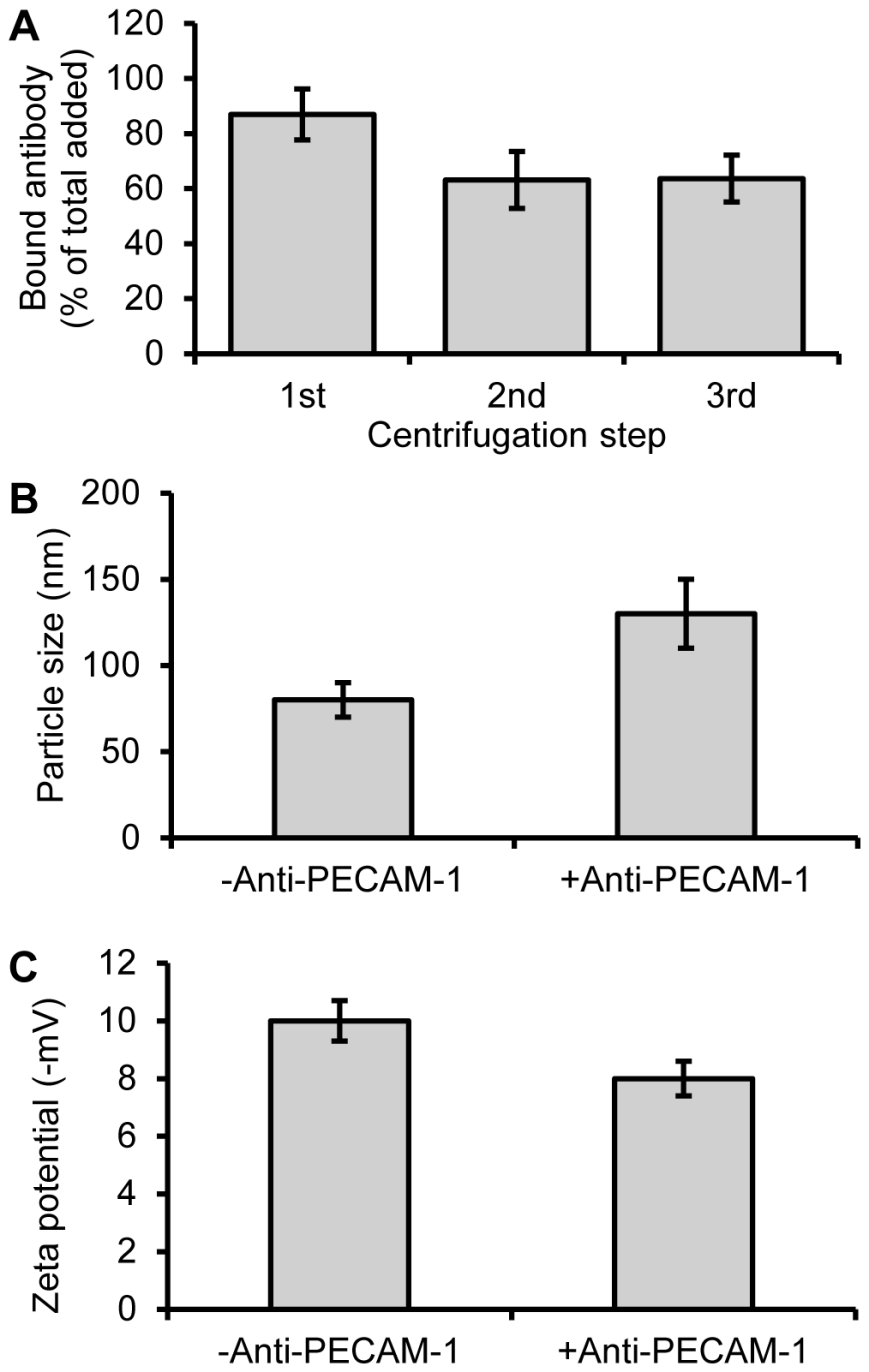
Surface modification of IONPs by anti-PECAM-1 antibody. Iron oxide nanoparticles were incubated with excess ^125^I labeled antibody and purified by centrifugation. The bound antibody was tested after each centrifugation (A). Size of IONPs before and after anti-PECAM-1 antibody surface modification (B). Zeta potential of IONPs before and after anti-PECAM-1 antibody surface modification (C). (N = 3, mean ± SD)

After removal of unbound antibody, the surface coverage was determined to be 63.6±8.4% (Figure 1A). Based on the primary nanoparticle size of 80 nm (Figure 1B), this corresponds to 19.1 µg antibody/mg of nanoparticle or 105 antibody molecules/nanoparticle. DLS measurements showed the size increased from 80 nm to 130 nm after addition of anti-PECAM-1 (Figure 1B), indicating uniform coating with slight aggregation, as the antibody size is ∼15 nm in length. The zeta potential of the nanoparticles decreased from −10 to −8 mV (Figure 1C).

### Stability of antibody-coated iron oxide nanoparticles in whole blood

**Figure 2:**
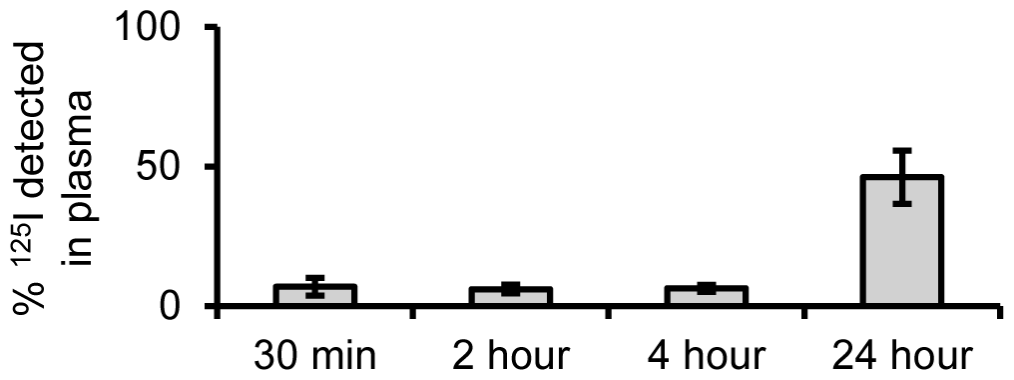
Antibody coating stability in whole blood. ^125^I labeled nanoparticles show minimal detachment of coating for up to 4 h, suggesting coating stability and targeting capability *in vivo*. (N = 3, mean ± SD)

Because anti-PECAM-1 IONPs and IgG IONPs were prepared by surface antibody adsorption, their stability in blood is very important for *in vivo* study. The ^125^I labeled IgG antibody exhibited minimal detachment from nanoparticles for up to 4 h at 37°C. At 4 h, only 6.4±1.2% of labeled antibody was detected in the heparin-treated blood after centrifugation. Between 4 and 24 h this increased to 46.2±9.5%, likely due to the antibody on the nanoparticle surface being replaced with higher affinity serum proteins (Figure 2). The insignificant coating loss over 4 h suggests these nanoparticle modifications will stay stable throughout the circulation life and time frame of *in vivo* experiments.

### PECAM-1 binding affinity to the human endothelial cell hCMEC/D3

**Figure 3:**
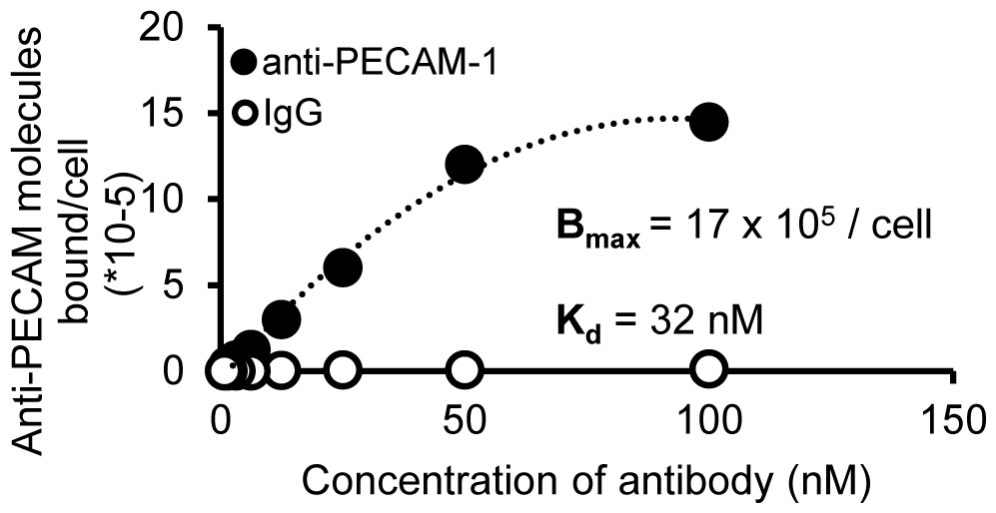
PECAM-1 antibody binding affinity to hCMEC/D3 cells. IgG exhibited undetectable levels of binding, whereas anti-PECAM-1 affinity was 32 nM, with a B_max_ of 17×10^5^ molecules/cell. (N = 3, mean ± SD)

hCMEC/D3 is a human BBB cell line developed in 2005. Previous research showed that it expressed PECAM-1 [24]. However, the binding affinity between hCMEC/D3 and PECAM-1 antibody was not known. Figure 3 shows that anti-PECAM-1, but not non-specific control IgG, adhered specifically to this BBB cell model. The predicted antibody saturation (B_max_) was determined to be 16.94×10^5^ molecules/cell, with a binding constant (K_D_) of 32 nM, which is relatively low compared with a designed PECAM-1 antibody (a paired monoclonal antibody) for vascular targeting, with reported affinities between 0.5–5 nM [30]. However, previous research showed that relatively low affinity antibodies boost brain uptake by transcytosis targeting [31]. In the next experiment, anti-PECAM-1 IONP flux across the human endothelial cell hCMEC/D3 was tested.

### Anti-PECAM-1 IONP flux and cell association using a hCMEC/D3 *in vitro* BBB model

**Figure 4:**
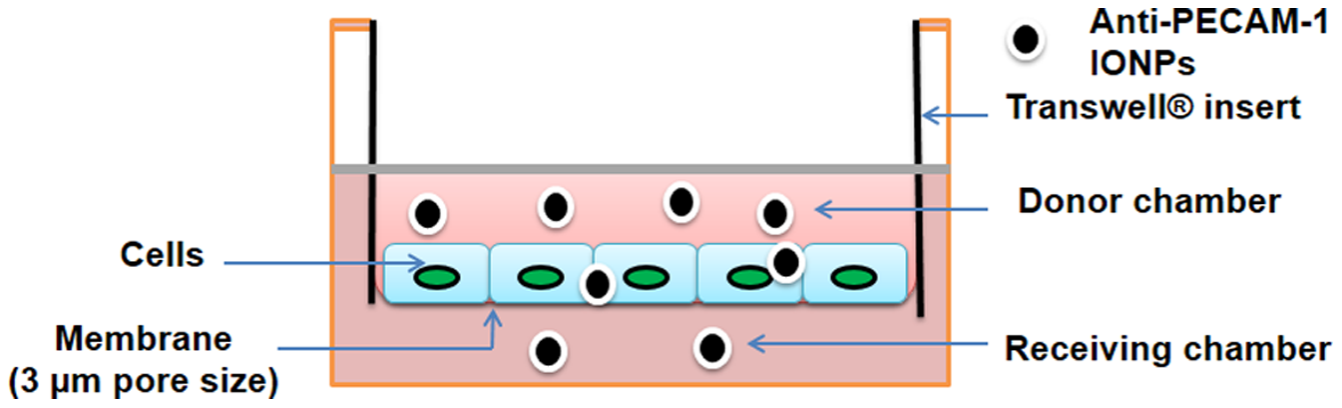
Schema of the Transwell® system used for flux studies.

The TEER of the hCMEC/D3 *in vitro* BBB model (Figure 4) was tested every other day after the cells were seeded. After 7-10 days, the resistances were >90 Ω/cm^2^, similar to previously reported [22]. The permeability coefficient of LY, the indication of paracellular flux, was 2.9±0.2×10^−6^ cm/s.

**Figure 5:**
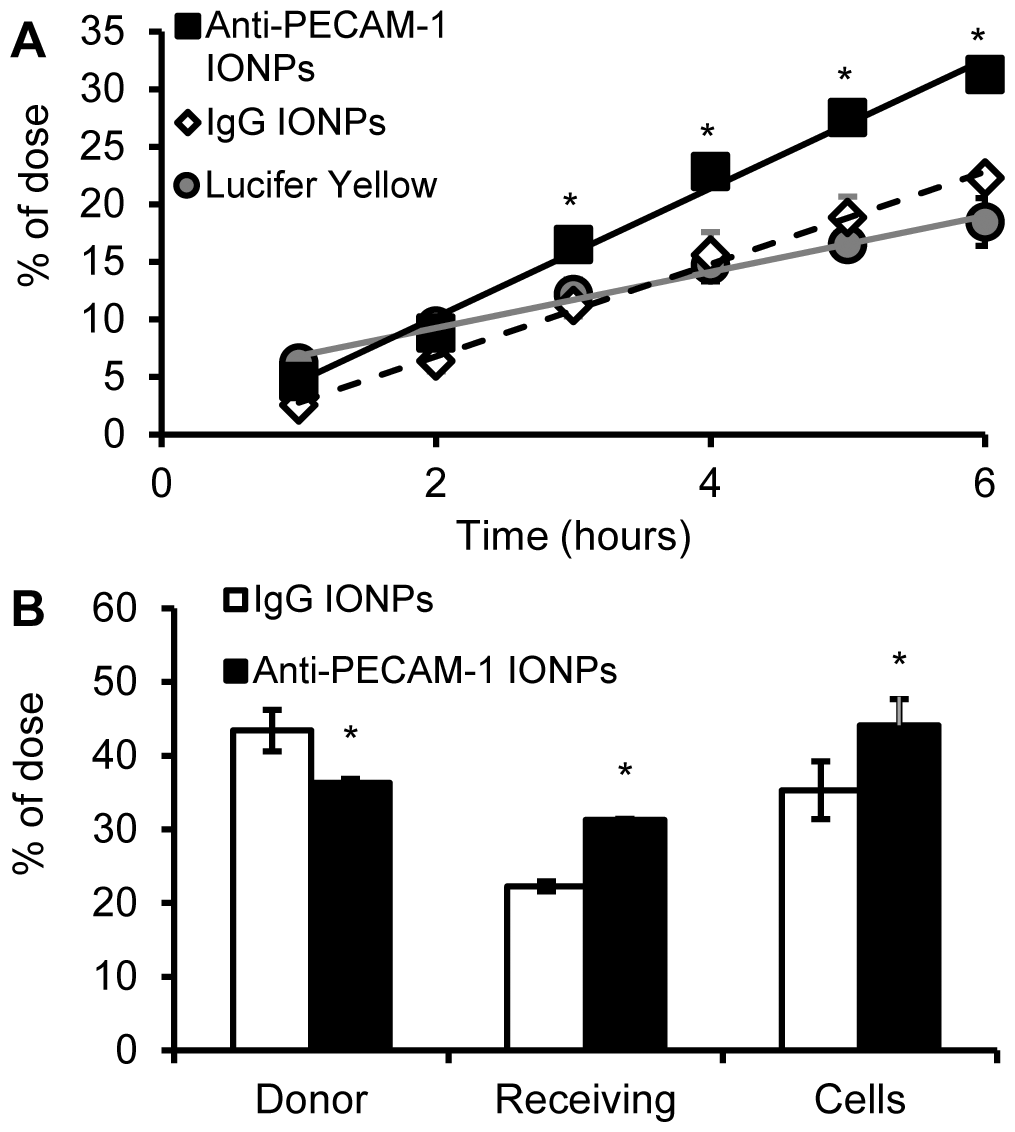
Anti-PECAM-1 IONP flux across hCMEC/D3 cells and cell association results. Anti-PECAM-1 IONP (0.05 mg/ml), IgG IONP (0.05 mg/ml) and LY (100 µM) flux across hCMEC/D3 cells for 6 h (A). Anti-PECAM-1 IONP and IgG IONP distribution in the donor and receiving chambers and hCMEC/D3 cells at 6 h (B). (N = 3, mean ± SD) * Significantly different compared to IgG IONPs.

Anti-PECAM-1 IONP flux was significantly higher than IgG IONPs and LY from 3 h to 6 h. The permeability coefficient after 6 h of anti-PECAM-1 IONPs was 6.7±0.2×10^−6^ cm/s, versus 4.8±0.2×10^−6^ cm/s for IgG IONPs, and 2.9±0.2×10^−6^ cm/s for LY (Figure 5A). After 6 h, 30% of anti-PECAM-1 IONPs was in the receiving chamber and ∼45% of anti-PECAM-1 IONPs was associated with the hCMEC/D3 cells, significantly higher than IgG IONPs (Figure 5B). PECAM-1 antibody significantly enhanced the flux of IONPs across the hCMEC/D3 monolayer *in vitro*. In the next experiment, anti-PECAM-1 IONP brain targeting, accumulation, and biodistribution were studied using Sprague Dawley rats.

### Anti-PECAM-1 IONP association and biodistribution in brain and peripheral organs

**Figure 6:**
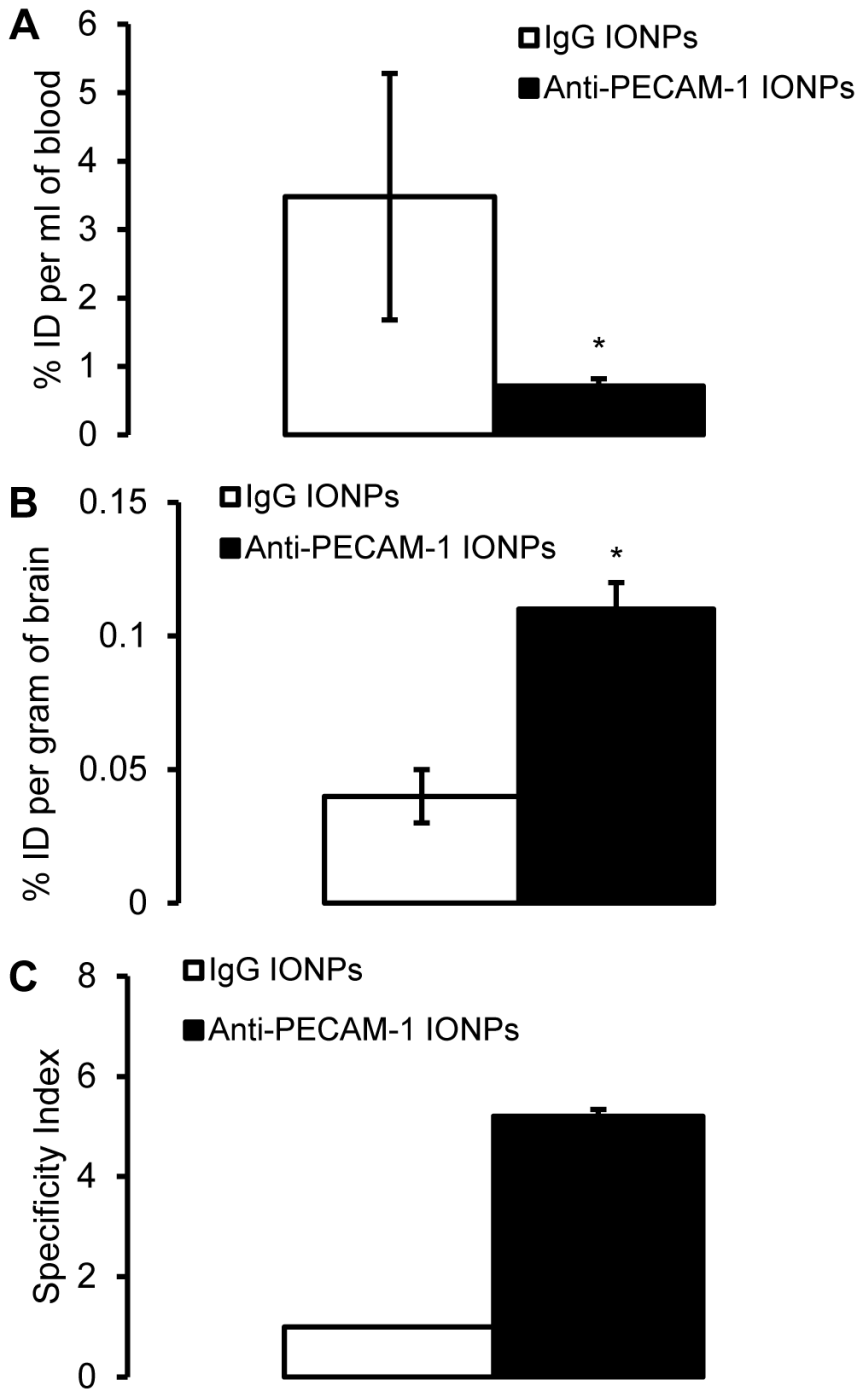
Percent of the anti-PECAM-1- and IgG IONPs dose in blood and brain association. The blood (A) and brain (B) levels of ^125^I labeled anti-PECAM-1-IONPs after intra-arterial infusion in rats, expressed as the percentage of injected dose (% Injected Dose (ID), 10 mg/kg). Specific tissue accumulation of anti-PECAM-1 IONPs compared with IgG IONPs in brain, calculated as the specificity index (SI). SI values above 1 represent specific targeting in an organ over IgG IONPs (C). * Significantly different compared to IgG IONPs. (N = 3, mean ± SD)

**Figure 7:**
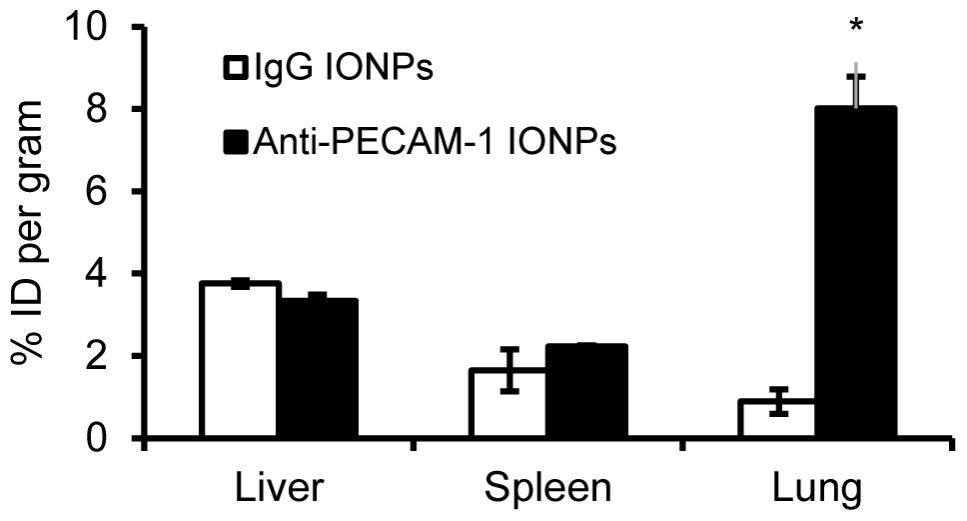
Biodistribution of IgG IONPs and anti-PECAM-1 IONPs in rats. The liver, spleen and lung levels of ^125^I labeled anti-PECAM-1 IONPs measured 10 min after intra-arterial infusion in rats, expressed as the percentage of injected dose (%ID, 10 mg/kg). * Significantly different compared to IgG IONPs. (N = 3, mean ± SD)

Using ^125^I tracing, we tested anti-PECAM-1 IONP targeting ability to brain and peripheral organs. As shown in figure 6A, the % dose per mL of blood in anti-PECAM-1 IONP treated rats was significantly lower than IgG IONP treatment, suggesting increased removal from blood and enhanced tissue accumulation. Ten min after infusion, 0.11±0.01% of the anti-PECAM-1 IONPs dose was associated with each gram of brain, which was significantly higher than anti-IgG IONPs (Figure 6B). The specificity index (the ratio between targeted and non targeted control) was calculated to test the anti-PECAM-1 IONP's brain targeting ability. Anti-PECAM-1 IONPs specificity in the brain was 5-fold higher than with IgG IONPs (Figure 6C). PECAM-1 targeting did not change anti-PECAM-1 IONP distribution in liver and spleen compared with IgG. However, anti-PECAM-1 IONP accumulation was significantly increased in the lungs (Figure 7).

### Effect of Anti-PECAM-1 IONPs on blood-brain barrier integrity

**Figure 8:**
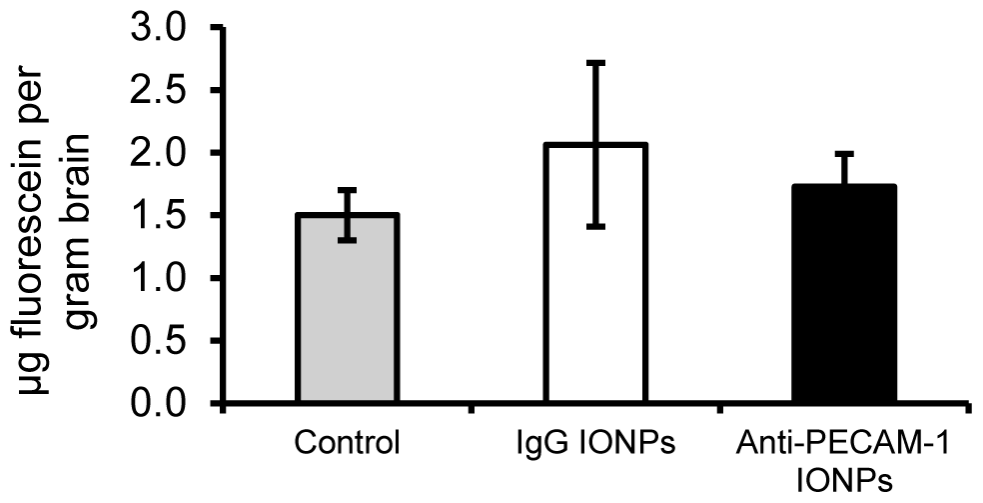
BBB permeability measured by fluorescein concentration 10-arterial infusion in rats. Rats received saline, 10/kg IgG IONPs, or 10 mg/kg anti-PECAM-1 IONPs and were terminated 10 min after infusion. (N = 3, mean ± SD)

Since anti-PECAM-1 IONP brain association was significantly increased, it was important to determine whether that changed the BBB permeability. Ten min after anti-PECAM-1 IONP injection the concentration of the BBB permeability marker, fluorescein in the brain, did not change significantly compared with the control and IgG IONP groups (Figure 8).

### Anti-PECAM-1 IONP distribution between brain capillary cells and brain parenchyma

**Figure 9:**
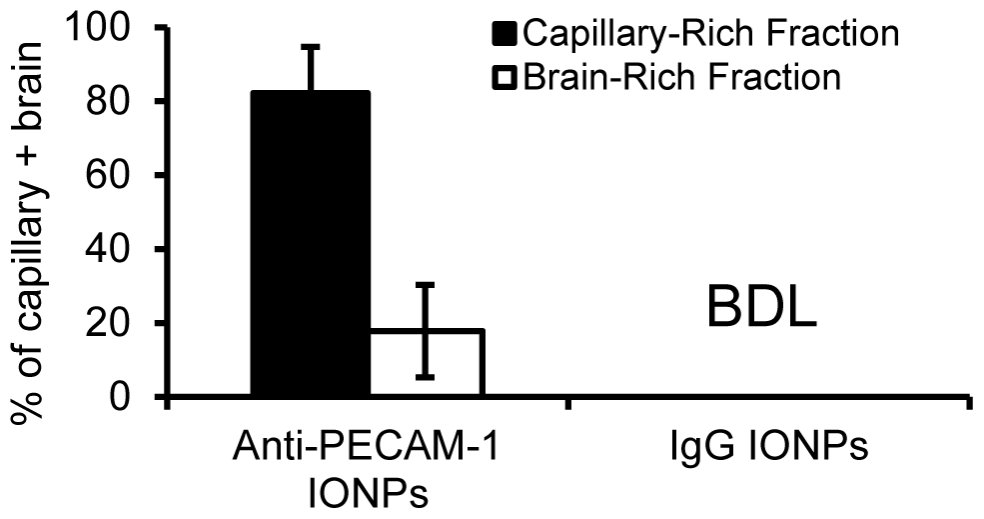
Capillary depletion results. The level of anti-PECAM-1 IONPs and IgG IONPs concentrations in the capillary-rich fraction and brain-rich fraction. (N = 3, mean ± SD). BDL: Below detectable limits.

For brain delivery systems, determination of whether or not they can enter the brain parenchyma is crucial. Our permeability results showed that anti-PECAM-1 IONPs did not alter BBB permeability. Understanding the anti-PECAM-1 IONP distribution between the brain capillary cells and parenchyma would provide evidence for transcellular flux. The capillary depletion results showed that 10 min after infusion, 82±12% of anti-PECAM-1 IONPs were associated with the capillary fraction and 17±12% of them entered the brain (Figure 9). The capillary depletion assay was also carried out with brain from IgG-IONPs treated rats. However, because of the low brain association of IgG IONPs, all radioactivity level readings were indistinguishable from background radioactivity.

## Discussion

The use of vasculature-targeting antibodies, especially against PECAM-1, has been utilized before for lung targeting, injury treatment, [32] and as tumor contrast agents [33]. Anti-PECAM-1 nanocarriers can internalize into the cell through cellular adhesion molecule (CAM)-mediated endocytosis [15], which provides the potential to target the BBB and increase nanocarrier flux across the BBB. Previous research showed that IONPs coupled with affinity moieties targeted to receptors, such as transferrin, can facilitate IONP flux across the BBB [34]. However, little is known whether anti-PECAM-1 surface-modified IONPs enhance brain flux across the BBB and change the distribution of IONPs between the BBB and brain parenchyma. In this study, we explored anti-PECAM-1 IONP targeting, transcytosis across the BBB, and biodistribution in the brain and peripheral organs.

We demonstrated that iron oxide nanoparticles can be sufficiently coupled with both IgG and anti-PECAM-1 using a non covalant physioabsorption strategy. It was determined that over 60% of the nanoparticle surface was coated with antibody (105 antibody molecules per nanoparticle). Previous research has demonstrated that clustering of the CAM through antibody binding can result in efficient internalization of anti-PECAM-1 nanoparticles, depending upon epitope of binding [15]. Using a non-covalant targeting strategy, it was expected that upon contact with serum proteins they would inactivate the targeting coating, lowering the treatment efficacy. Contrary to this, it was seen that the coating on these nanoparticles stays intact for up to 4 h, vastly longer than the circulation half life of up to 2 h for uncoated particles [35]. The IgG IONPs and anti-PECAM-1 we developed have desirable properties to investigate BBB targeting and flux *in vitro* and *in vivo*.

In our *in vitro* flux study, LY showed higher flux at 1 h compared with anti-PECAM-1 IONPs and IgG IONPs. At 2 h, the fluxes of LY, anti-PECAM-1 IONPs and IgG IONPs were similar. After 2 h, anti-PECAM-1 IONP flux was significantly higher than LY and IgG IONPs. These results provided evidence that the paracellular pathway was not the major pathway for anti-PECAM-1 IONPs flux. Anti-PECAM-1 IONPs use a different mechanism of flux across the BBB compared with IgG IONPs. There are multiple pathways for internalization involving vesicles <300 nm in diameter. Clathrin- and caveolea-mediated endocytosis are the two major pathways for nanoparticle internalization [36], [37], [38], [39]. IgG IONPs are likely to be taken up through these pathways. Limited transcytosis was observed in our study, which was consistent with a previous study in which the flux of three different surface-charged IONPs was studied across a human BBB model *in vitro*. Very limited flux was observed over 25 h [10]. On the other hand, clustered PECAM-1 can be internalized by a novel endocytic pathway, CAM endocytosis [15], which was distinct from clathrin and caveolin-mediated endocyosis [40], [41]. Our *in vitro* flux study showed that PECAM-1 antibody surface modification significantly improved BBB targeting and flux across the BBB. Previous research showed that intercellular adhesion molecule 1(ICAM-1)-targeted nanocarriers, which also use the CAM-endocytosis pathway, provide considerable promise to enhance delivery of larger multivalent carriers to the CNS [25]. Another study also showed that anti-PECAM-1 nanocarriers demostrated significantly higher brain association [42]. However, they analyzed whole brain tissue including the BBB, brain parenchyma and the blood in the brain vessels. The present study provides a better understanding of the assocation and flux of anti-PECAM-1 nanoparticles across the BBB *in vitro* and *in vivo*.

In our *in vivo* study, anti-PECAM-1 IONPs did not change BBB permeability, further suggesting that anti-PECAM-1 IONPs crossed the BBB through a transcellular pathway rather than a paracellular pathway. We are not aware of any reports on how anti-PECAM-1 IONPs influence BBB permeability. However, previous research showed that anti-PECAM nanocarriers did not change endothelial monolayer integrity compared with a IgG nanocarrier [42]. The lack of anti-PECAM IONP increased BBB permeability decreases its potential adverse effects related to the BBB.

The level of anti-PECAM-1 IONP in the blood was significantly lower than IgG IONP, suggesting enhanced tissue accumulation. The results were consistent with anti-PECAM-1 IONP brain association results. Compared with IgG IONP, anti-PECAM-1 IONP significantly increased IONP brain association 10 min after infusion. There was 0.11±0.01% of the dose associated with a gram of brain tissue 10 min after infusion. For comparison, the brain uptake of morphine, a neuroactive lipid soluble small molecule, is 0.0081±0.001% of the dose/g rat brain [43]. The uptake of anti-PECAM-1 IONPs was higher than morphine, a regularly administered neuroactive small molecule with the capability of crossing the BBB. This demonstrates the potential application of PECAM-1 antibody for brain delivery. The transferrin receptor is the most studied targeting receptor for brain uptake [44]. Most previous studies of transferrin-surface-modified nanoparticles focused on the improvement of diagnosis and therapeutic effects rather than brain uptake [34], [45]. The brain delivery of the transferrin ligand was less than 0.3% of the dose using a healthy animal model [46]. A recent study compared ICAM-1 antibody and transferrin-surface-modified nanocarriers for brain targeting. It was found that they are both effective, but transferrin showed more advantages on smaller conjugates and ICAM-1 worked better for larger multivalent carriers [24]. In our study anti-PECAM-1 IONPs showed a similar specificity index as previously reported anti-ICAM-1 nanocarriers [25]. We expected PECAM-1 antibody would show similar brain targeting as ICAM-1, however, more research needs to be done to compare PECAM-1, ICAM-1 and transferrin for brain targeting. Furthermore, the actual extent of transferrin transcytosis is still unknown. Some studies showed that only a miniscule amount of transferrin was trancytosed across the brain capillary cells and accumulated in the brain [47], [48]. Our *in vitro* results provide evidence that anti-PECAM-1 IONPs can transcytose across a human BBB monolayer *in vitro* model.

We investigated the distribution between the BBB and brain parenchyma using the capillary depletion method to better characterize anti-PECAM-1 IONP transcytosis *in vivo*. Our results showed that 10 min after intra-arterial infusioin, 17±12% of anti-PECAM-1 IONPs crossed the BBB and associated with brain parenchyma. However, the majority of anti-PECAM-1 IONPs was still associated with the BBB cells. CAM-mediated endocytosis is a relatively slow process. One study investigated anti-PECAM nanocarrier internalization into human endothelial cells over time. After 15 minutes, 20% of anti-PECAM nanocarrier was internalized. However, there is little known about anti-PECAM-1 transcytosis [42]. Our *in vitro* flux study showed that 4.5% of anti-PECAM-1 IONPs flux cross the BBB monolayer over 1 h. Our studies demonstrated the potential of anti-PECAM-1 IONPs to cross the BBB *in vitro* and *in vivo*. However, a longer time point study is required to better understand how effectively anti-PECAM-1 enhances flux across the BBB.

The biodistribution results of anti-PECAM-1 IONPs demostrated that PECAM-1 antibody did not increase anti-PECAM-1 IONP accumulation in the liver or spleen 10 min after injection. More study at longer time points is needed to fully understand the disposition of anti-PECAM-1 IONPs in the liver and spleen. However, PECAM-1 antibody significantly increased the IONP accumulation in the lung. This result was consistent with previous reports that an anti-PECAM-1 nanocarrier is a good candidate for pulmonary targeting [49], [50]. This is due to the massive surface area provided by lung capillary beds. For brain targeted delivery, high accumulation in the lung has potential to cause side effects there. However, we can take advantage of this property for certain diseases. For example, about 15–20% of patients with non-small cell lung cancer (NSCLC) develop brain metastasis [51]. Anti-PECAM-1 nanocarriers can target lung and brain simultaneously and be taken up through the CAM-mediated endocytosis pathway [42]. Furthermore, recent clinical research showed that PECAM-1 could be a potential prognostic factor and a novel therapeutic target for the effective treatment of NSCLC [52]. Therefore, anti-PECAM-1 IONPs have potential to be used to target lung and NSCLC, while treating potential brain metastasis. More research needs to be conducted to test anti-PECAM-1 IONPs in a brain metastasis model and investigate how they associate with the blood tumor barrier and tumor cells.

## Conclusions

This work demostrated that anti-PECAM-1-modified IONPs enhance flux across the BBB *in vitro* and *in vivo*, which holds promise to deliver IONPs or other therapeutic agents to the CNS without compromising BBB permeability. This effect was a result of both the capacity of anti-PECAM-1 IONPs to target the BBB and the ability to transcytose across it into the brain. Meanwhile, anti-PECAM-1 IONPs demonstrated increased lung accumulation, which provides the potential to simultaneously target lung and lung cancer derived-brain metastasis. Future studies investigating anti-PECAM-1 IONPs using a lung cancer brain metastasis model *in vivo* should provide evidence about how anti-PECAM-1 IONPs associate with the blood tumor barrier and metastatic brain tumor. Anti-PECAM-1 IONPs have great potential to be employed in the diagnosis and therapy of CNS diseases such as NSCLC-originating brain metastasis.
